# Relationship between bone mineral density and hyperuricemia in obesity: A cross-sectional study

**DOI:** 10.3389/fendo.2023.1108475

**Published:** 2023-03-28

**Authors:** Yi Zhang, Min Tan, Boyu Liu, Manxia Zeng, You Zhou, Mengru Zhang, Yikai Wang, Jing Wu, Min Wang

**Affiliations:** ^1^ Department of Endocrinology, Xiangya Hospital, Central South University, Changsha, Hunan, China; ^2^ Department of Orthopaedics, Xiangya Hospital, Central South University, Changsha, Hunan, China; ^3^ National Clinical Research Center for Geriatric Disorders, Xiangya Hospital, Changsha, Hunan, China; ^4^ Department of Hyperbaric Oxygen, People’s Hospital of Hunan Province, Hunan Normal University, Changsha, Hunan, China; ^5^ Department of Endocrinology, Yiyang Central Hospital, Yiyang, Hunan, China; ^6^ Hunan Engineering Research Center for Obesity and its Metabolic Complications, Xiangya Hospital, Central South University, Changsha, Hunan, China

**Keywords:** bone mineral density, hyperuricemia, obesity, cross-sectional, uric acid

## Abstract

**Background:**

Obesity is an increasingly severe global public health issue. This study aims to estimate the cross-sectional association between bone mineral density (BMD) and hyperuricemia (HU) in obesity.

**Method:**

A total of 275 obese subjects (126 men and 149 women) participated in this cross-sectional study. Obesity was diagnosed as body mass index (BMI) ≥28 kg/m^2^, whereas HU was defined as the blood uric acid level of 416 μmol/L in men and 360 μmol/L in women. The BMD of the lumbar spine and right hip was measured by dual-energy X-ray absorptiometry (DXA). The multivariable logistic regressions were employed to examine the relationship between BMD and HU in obesity, with the adjustment of gender, age, fasting blood glucose, fasting insulin, homeostasis model assessment of insulin resistance (HOMA-IR), cholesterol, triglycerides, low-density lipoprotein, high-density lipoprotein, creatinine, blood urea nitrogen, high-sensitivity C-reactive protein (hs-CRP), cigarette smoking, and alcohol drinking status.

**Result:**

The overall prevalence of HU was 66.9% in this obese population. The mean age and BMI of this population were 27.9 ± 9.9 years and 35.2 ± 5.2 kg/m^2^, respectively. The multivariable-adjusted OR (the highest *vs.* lowest BMD quartile) demonstrated a negative relationship between BMD and HU in total (OR = 0.415, 95%CI: 0.182–0.946; p = 0.036), L1 (OR = 0.305, 95%CI: 0.127–0.730; p = 0.008), L2 (OR = 0.405, 95%CI: 0.177–0.925; p = 0.032), and L3 (OR = 0.368, 95%CI: 0.159–0.851; p = 0.020) lumbar vertebrae. In the subgroup analysis for the male population, the BMD was also negatively associated with HU in total (OR = 0.077, 95%CI: 0.014–0.427; p = 0.003), L1 (OR = 0.019, 95%CI: 0.002–0.206; p = 0.001), L2 (OR = 0.161, 95%CI: 0.034–0.767; p = 0.022), L3 (OR = 0.186, 95%CI: 0.041–0.858; p = 0.031), and L4 (OR = 0.231, 95%CI: 0.056–0.948; p = 0.042) lumbar vertebrae. However, such findings did not exist in women. In addition, there was no significant relationship between hip BMD and HU in obesity.

**Conclusion:**

Our results showed that the lumbar BMD was negatively associated with HU in obesity. However, such findings only existed in men, rather than women. In addition, no significant relationship between hip BMD and HU existed in obesity. Due to the limited sample size and nature of the cross-sectional design, further large prospective studies are still needed to clarify the issues.

## Introduction

Obesity is an increasingly severe clinical and public health issue. Unfortunately, more than 100 million patients suffer from obesity worldwide (1). More importantly, obesity has become a major public health concern in China. It was estimated that more than half of Chinese adults are either overweight or obese in a recent national survey (2, 3). Emerging evidence indicated that obesity is associated with a higher risk of hyperuricemia (HU) (4). Uric acid is an end product of purine metabolism in the human body, and HU is always recognized as the precursor of gout (5). In addition, HU is also considered to be related to metabolic syndromes/indices (3, 6), renal injury (7), inflammation, and endothelial dysfunction (8) in obesity. Therefore, the identification of modifiable factors for HU appears to be an important step in the clinical management of obesity.

Generally speaking, bone mineral density (BMD) is an important and common clinical indicator for the diagnosis of osteoporosis (9). As far as we know, a large number of studies have examined the association between HU and BMD. However, no final conclusion can be obtained (9–20). The potential reasons for such discrepancy may be attributed to the variety of population characteristics, including genetic background, age, body fat proportion or confounders adjusted, and different lifestyle factors (14). Therefore, it is significant and necessary to consider the issue in different sub-populations. Obesity is a health issue with special clinical characteristics. Importantly, a causal relationship between obesity and lower BMD was confirmed in a two-sample Mendelian randomization study recently (21). Indeed, increased marrow adiposity is attributed to the shift from osteogenic to adipogenic differentiation of bone marrow mesenchymal stem cells (22). However, obesity is also positively associated with HU (23, 24). Experimental evidence did suggest that purine catabolism in adipose tissue could be enhanced in obesity (25). It is interesting and necessary to reveal how BMD is involved in HU in the context of obesity. Therefore, this study was performed to investigate the relationship between BMD and HU in the obese population.

## Materials and methods

### Study population

This cross-sectional study was reviewed and approved by the Medical Ethics Committee of Xiangya Hospital, Central South University (202103043). A total of 275 obese patients (126 men and 149 women) were recruited from Xiangya Hospital (from August 2019 to December 2021). The inclusion criteria were as follows: 1) BMI ≥ 28 kg/m^2^, 2) available completed clinical data, and 3) patients agreeing to participate in the study. The exclusion criteria were as follows: 1) postmenopausal women; 2) bone disease (new fractures and malignancies), severe hepatic and renal insufficiency, thyroid and parathyroid disorders, and some other diseases involved in bone metabolism; 3) long-term drug users with affected bone or uric acid metabolism (e.g., glucocorticoids, anticoagulants, thyroid hormones, proton pump inhibitors, antiepileptics, allopurinol, and benzbromarone); 4) those who have undergone iodine, barium, or nuclear medicine isotope tests in the past week (bone scan, kidney scan, PET-CT, enhanced CT, etc.); 5) subjects with implanted materials that affect BMD assessment (bone cement, surgical nails, steel stents, metal implants, pacemaker lead wires, etc.).

### Blood biochemistry

The blood biochemical indices of subjects who fasted for 8–12 h were obtained. The fasting venous blood was collected in the morning. Blood uric acid, fasting blood glucose, fasting insulin, cholesterol, triglyceride, low-density lipoprotein, high-density lipoprotein, serum creatinine, blood urea nitrogen, and high-sensitivity C-reactive protein (hs-CRP) were collected and recorded. Moreover, the homeostasis model assessment of insulin resistance (HOMA-IR) was also calculated. The blood biochemical indices were tested using the automatic biochemical analyzer. Uric acid was determined by the uricase–peroxidase method. Fasting blood glucose and insulin were determined by hexokinase and chemiluminescence methods. Cholesterol was determined by the enzyme method, triglyceride was determined by the GPO-POD method, and low-density lipoprotein and high-density lipoprotein were determined by a direct method. Moreover, creatinine was determined by the basic picric acid method, urea nitrogen was determined by the glutamate dehydrogenase method, and hs-CRP was determined by the immunoturbidimetric method. HOMA-IR was calculated by the following formula: fasting blood glucose (mmol/L) * fasting insulin (mIU/L)/22.5. HU was defined as a blood uric acid level of 416 μmol/L in men and 360 μmol/L in women.

### Measurement of bone mineral density

**Figure 1:**
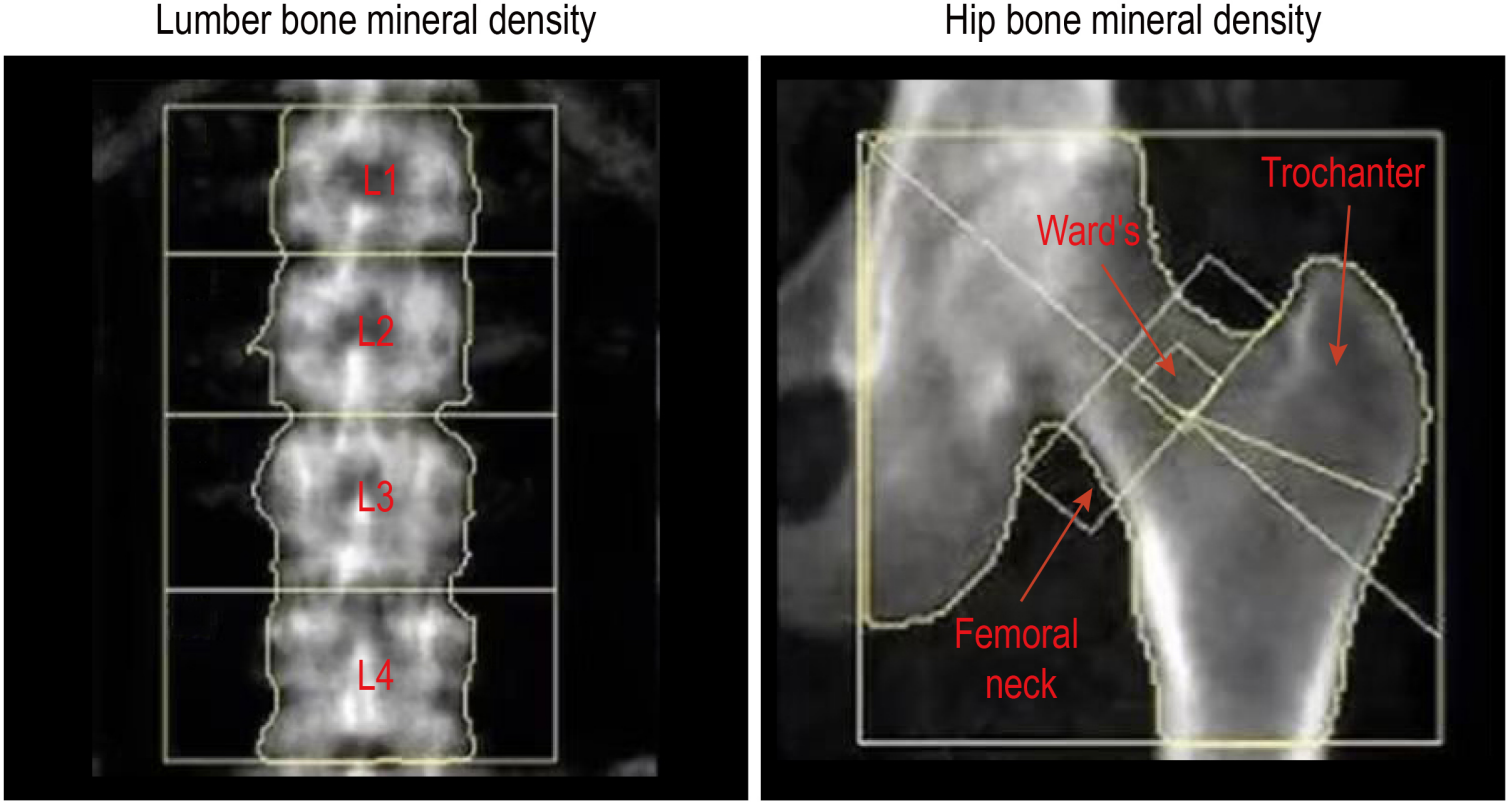
The assessment of bone mineral density in the lumbar spine and hip.

The BMD of the lumbar spine and right hip was measured by dual-energy X-ray absorptiometry (DXA) (U.S. Lunar), which is commonly used in clinical and scientific research due to its small repeatability and diagnostic error. The BMD was measured by the same experienced senior doctor, and quality control testing was carried out properly. Lumbar spine BMD measurements included the first, second, third, and fourth lumbar vertebrae (L1 to L4) and total lumbar vertebra, whereas right hip BMD measurements included the femoral neck, trochanter, Ward’s triangle, and total hip (**Figure 1**). Anatomically,. Ward’s triangle is a fragile triangular area formed by the cross of the femoral neck between the medial pressure bone trabecula, lateral tension bone trabecula, and intertrochanteric line of the femoral neck (a common site for fracture). The BMD levels were expressed as BMD values (g/cm^2^) directly.

### General information collection

The gender, age, height, and weight of the included subjects were collected and recorded. The body mass index (BMI) was calculated by the following formula: BMI (kg/m^2^) = weight (kg)/height (m)^2^. Both cigarette smoking and alcohol drinking status were evaluated by the questions “Do you smoke/drink? Yes/No”.

### Statistical analysis

The SPSS 23.0 software was utilized for the analysis. The normal distribution data are expressed by means and standard deviation, whereas the skewness distribution data are expressed by median and interquartile spacing. The classified data are expressed as percentage. The t-test was employed for normal distribution data, whereas the chi-square test was used for classified data. The rank sum test was used for skewed distribution data. Moreover, multiple logistic regression was used for correlation analysis. The differences in general data, blood biochemical indicators, and BMD between obese men and women were also compared. The BMD and HU served as exposure and outcome, respectively. The BMD was divided into quartiles from low to high, with the lowest quartile as the reference. The following covariates were adjusted: gender, age, fasting blood glucose, fasting insulin, HOMA-IR, cholesterol, triglycerides, low-density lipoprotein, high-density lipoprotein, creatinine, blood urea nitrogen, hs-CRP, cigarette smoking, and alcohol drinking status. p < 0.05 was considered statistically significant.

## Results

### The main characteristics of the obese population

The main characteristics of the obese population are presented in **Table 1**. A total of 275 subjects were included in our study: 126 and 149 were men and women, respectively. The mean age and BMI of this population were 27.9 ± 9.9 years and 35.2 ± 5.2 kg/m^2^, respectively. The BMI, uric acid, triglyceride, HDL cholesterol, creatinine, cigarette smoking, alcohol drinking, total hip BMD, femoral neck BMD, and trochanter BMD were all significantly different between men and women.

**Table 1:** The main characteristics of the obese population.

Characteristics	All	Male	Female	p-Value
Participants (n)	275	126	149	–
Age (years)	27.9 ± 9.9	28.1 ± 10.6	27.7 ± 9.2	0.726
BMI (kg/m^2^)	35.2 ± 5.2	36.9 ± 5.5	33.7 ± 4.4	**<0.001**
Uric acid (μmol/L)	444.2 ± 118.6	500.5 ± 121.0	396.6 ± 93.2	**<0.001**
Hyperuricemia (yes, %)	66.9	65.9	67.8	0.737
FBG (mmol/L)	5.41 (1.10)	5.37 (1.10)	5.43 (1.00)	0.736
FINS (mIU/L)	20.9 (15.7)	20.8 (15.6)	20.9 (15.7)	0.211
HOMA-IR	5.14 (4.33)	5.39 (4.68)	5.05 (4.30)	0.319
Triglyceride (mmol/L)	1.71 (1.36)	2.06 (1.40)	1.50 (1.11)	**<0.001**
Cholesterol (mmol/L)	5.07 ± 1.02	5.13 ± 1.14	5.02 ± 0.91	0.383
LDL cholesterol (mmol/L)	3.29 ± 0.72	3.30 ± 0.77	3.28 ± 0.68	0.748
HDL cholesterol (mmol/L)	1.11 ± 0.24	1.04 ± 0.22	1.16 ± 0.24	**<0.001**
Creatinine (μmol/L)	75.1 ± 19.5	85.7 ± 23.2	66.0 ± 8.4	**<0.001**
BUN (mmol/L)	4.44 ± 2.11	4.55 ± 1.32	4.34 ± 2.60	0.403
hs-CRP (mg/L)	3.24 (3.62)	2.90 (3.68)	3.44 (3.47)	0.227
Cigarette smoking (yes, %)	23.3	40.5	8.7	**<0.001**
Alcohol drinking (yes, %)	14.5	26.2	4.7	**<0.001**
Total hip BMD (g/cm^2^)	1.033 ± 0.139	1.073 ± 0.133	1.001 ± 0.136	**<0.001**
Femoral neck BMD (g/cm^2^)	0.901 ± 0.121	0.940 ± 0.124	0.870 ± 0.110	**<0.001**
Trochanter BMD (g/cm^2^)	0.762 ± 0.103	0.783 ± 0.106	0.745 ± 0.098	**0.009**
Ward’s triangle BMD (g/cm^2^)	0.853 ± 0.179	0.870 ± 0.186	0.839 ± 0.174	0.229
Total lumbar BMD (g/cm^2^)	1.041 ± 0.120	1.033 ± 0.127	1.047 ± 0.113	0.304
L1 BMD (g/cm^2^)	0.990 ± 0.123	0.991 ± 0.122	0.990 ± 0.124	0.947
L2 BMD (g/cm^2^)	1.044 ± 0.124	1.038 ± 0.129	1.048 ± 0.121	0.510
L3 BMD (g/cm^2^)	1.073 ± 0.134	1.056 ± 0.139	1.087 ± 0.129	0.056
L4 BMD (g/cm^2^)	1.047 ± 0.130	1.036 ± 0.144	1.056 ± 0.118	0.197

The p-value is derived from comparison between men and women.

BMI, body mass index; FBG, fasting blood glucose; FINS, fasting insulin; HOMA-IR, homeostasis model assessment of insulin resistance; LDL cholesterol, low-density lipoprotein cholesterol; HDL cholesterol, high-density lipoprotein cholesterol; BUN, blood urea nitrogen; hs-CRP, high-sensitivity C-reactive protein; BMD, bone mineral density; L1, first lumbar vertebra; L2, second lumbar vertebra; L3, third lumbar vertebra; L4, fourth lumbar vertebra. The bold values indicate the datawith a significant P value (P<0.05).

### The relationship between lumbar BMD and hyperuricemia in obesity

The results of the relationship between lumbar BMD and HU in obesity are presented in **Table 2**. The multivariable-adjusted OR (the highest *vs.* lowest BMD quartile) demonstrated a negative relationship between BMD and HU in total (OR = 0.415, 95%CI: 0.182–0.946; p = 0.036), L1 (OR = 0.305, 95%CI: 0.127–0.730; p = 0.008), L2 (OR = 0.405, 95%CI: 0.177–0.925; p = 0.032), and L3 (OR = 0.368, 95%CI: 0.159–0.851; p = 0.020) lumbar vertebrae. In the subgroup analysis for the men population, the BMD was also negatively associated with HU in total (OR = 0.077, 95%CI: 0.014–0.427; p = 0.003), L1 (OR = 0.019, 95%CI: 0.002–0.206; p = 0.001), L2 (OR = 0.161, 95%CI: 0.034–0.767; p = 0.022), L3 (OR = 0.186, 95%CI: 0.041–0.858; p = 0.031), and L4 (OR = 0.231, 95%CI: 0.056–0.948; p = 0.042) lumbar vertebrae. On the contrary, no significant relationship between lumbar BMD and HU was obtained in women.

**Table 2:** Multivariable-adjusted ORs of hyperuricemia according to lumbar bone mineral density level in obese subjects.

	Total		Male		Female	
	Multivariable-adjusted OR	p-Value	Multivariable-adjusted OR	p-Value	Multivariable-adjusted OR	p-Value
Total lumbar						
Quartile 1	1	/	1	/	1	/
Quartile 2	1.064 (0.437–2.593)	0.891	0.146 (0.022–0.953)	**0.044**	2.510 (0.734–8.586)	0.143
Quartile 3	0.645 (0.279–1.492)	0.305	0.099 (0.017–0.569)	**0.010**	1.277 (0.403–4.047)	0.678
Quartile 4	0.415 (0.182–0.946)	**0.036**	0.077 (0.014–0.427)	**0.003**	0.984 (0.320–3.026)	0.978
L1						
Quartile 1	1	/	1	/	1	/
Quartile 2	0.588 (0.243–1.424)	0.239	0.062 (0.006–0.643)	**0.020**	0.788 (0.248–2.502)	0.686
Quartile 3	0.464 (0.187–1.152)	0.098	0.036 (0.003–0.400)	**0.007**	0.798 (0.245–2.600)	0.709
Quartile 4	0.305 (0.127–0.730)	**0.008**	0.019 (0.002–0.206)	**0.001**	0.854 (0.264–2.767)	0.793
L2						
Quartile 1	1	/	1	/	1	/
Quartile 2	0.916 (0.387–2.169)	0.842	0.250 (0.049–1.278)	0.096	1.240 (0.382–4.026)	0.720
Quartile 3	0.667 (0.288–1.542)	0.343	0.160 (0.031–0.818)	**0.028**	1.081 (0.347–3.366)	0.893
Quartile 4	0.405 (0.177–0.925)	**0.032**	0.161 (0.034–0.767)	**0.022**	0.690 (0.221–2.157)	0.524
L3						
Quartile 1	1	/	1	/	1	/
Quartile 2	0.634 (0.263–1.526)	0.309	0.321 (0.064–1.612)	0.168	2.013 (0.625–6.481)	0.241
Quartile 3	0.532 (0.221–1.278)	0.158	0.206 (0.044–0.962)	**0.045**	0.916 (0.288–2.912)	0.881
Quartile 4	0.368 (0.159–0.851)	**0.020**	0.186 (0.041–0.858)	**0.031**	1.461 (0.463–4.617)	0.518
L4						
Quartile 1	1	/	1	/	1	/
Quartile 2	1.392 (0.581–3.336)	0.459	0.767 (0.160–3.675)	0.740	4.752 (1.340–16.855)	**0.016**
Quartile 3	0.966 (0.417–2.238)	0.936	0.377 (0.086–1.649)	0.195	1.525 (0.453–5.132)	0.496
Quartile 4	0.538 (0.238–1.220)	0.138	0.231 (0.056–0.948)	**0.042**	1.400 (0.456–4.299)	0.557

The multivariable model was adjusted for age (continuous data), BMI (continuous data), gender (male, female), FBG (continuous data), FINS (continuous data), HOMA-IR (continuous data), triglyceride (continuous data), cholesterol (continuous data), LDL cholesterol (continuous data), HDL cholesterol (continuous data), creatinine (continuous data), BUN (continuous data), hs-CRP (continuous data), cigarette smoking (yes or no), and alcohol drinking (yes or no).

BMI, body mass index; FBG, fasting blood glucose; FINS, fasting insulin; HOMA-IR, homeostasis model assessment of insulin resistance; LDL cholesterol, low-density lipoprotein cholesterol; HDL cholesterol, high-density lipoprotein cholesterol; BUN, blood urea nitrogen; hs-CRP, high-sensitivity C-reactive protein; L1, first lumbar vertebra; L2, second lumbar vertebra; L3, third lumbar vertebra; L4, fourth lumbar vertebra. The bold values indicate the data with a significant P value (P<0.05).

### The relationship between hip BMD and hyperuricemia in obesity

The results of the relationship between hip BMD and HU in obesity are presented in **Table 3**. The multivariable-adjusted OR (the highest *vs.* lowest BMD quartile) demonstrated no significant relationship between BMD and HU in the total hip (OR = 0.996, 95%CI: 0.365–2.718; p = 0.994), femoral neck (OR = 1.086, 95%CI: 0.377–3.129; p = 0.879), trochanter (OR = 0.986, 95%CI: 0.375–2.590; p = 0.976), and Ward’s triangle (OR = 1.965, 95%CI: 0.695–5.556; p = 0.203). In the subgroup analysis, no significant relationship between BMD and HU was obtained in the male and female populations.

**Table 3:** Multivariable-adjusted ORs of hyperuricemia according to hip bone mineral density level in obese subjects.

	Total		Male		Female	
	Multivariable-adjusted OR	p-Value	Multivariable-adjusted OR	p-Value	Multivariable-adjusted OR	p-Value
Total hip						
Quartile 1	1	/	1	/	1	/
Quartile 2	1.774 (0.701–4.489)	0.226	3.457 (0.453–26.405)	0.232	2.828 (0.740–10.799)	0.128
Quartile 3	2.095 (0.804–5.455)	0.130	0.425 (0.071–2.547)	0.349	1.758 (0.452–6.840)	0.416
Quartile 4	0.996 (0.365–2.718)	0.994	0.523 (0.076–3.614)	0.511	2.002 (0.521–7.688)	0.312
Femoral neck						
Quartile 1	1	/	1	/	1	/
Quartile 2	1.079 (0.437–2.669)	0.869	0.366 (0.056–2.416)	0.297	1.570 (0.426–5.784)	0.498
Quartile 3	0.852 (0.326–2.229)	0.745	0.286 (0.042–1.927)	0.198	1.690 (0.448–6.378)	0.439
Quartile 4	1.086 (0.377–3.129)	0.879	0.126 (0.015–1.056)	0.056	1.660 (0.403–6.845)	0.483
Trochanter						
Quartile 1	1	/	1	/	1	/
Quartile 2	0.954 (0.390–2.332)	0.918	0.119 (0.016–0.883)	**0.037**	1.382 (0.393–4.864)	0.614
Quartile 3	1.376 (0.524–3.615)	0.517	0.359 (0.072–1.790)	0.212	1.491 (0.403–5.514)	0.549
Quartile 4	0.986 (0.375–2.590)	0.976	0.325 (0.058–1.828)	0.202	0.908 (0.241–3.426)	0.887
Ward’s						
Quartile 1	1	/	1	/	1	/
Quartile 2	1.551 (0.610–3.945)	0.357	1.031 (0.153–6.921)	0.975	1.912 (0.538–6.797)	0.316
Quartile 3	1.531 (0.581–4.031)	0.389	0.974 (0.166–5.715)	0.976	2.352 (0.576–9.600)	0.233
Quartile 4	1.965 (0.695–5.556)	0.203	0.920 (0.134–6.319)	0.933	1.973 (0.439–8.868)	0.375

The multivariable model was adjusted for age (continuous data), BMI (continuous data), gender (male, female), FBG (continuous data), FINS (continuous data), HOMA-IR (continuous data), Triglyceride (continuous data), cholesterol (continuous data), LDL cholesterol (continuous data), HDL cholesterol (continuous data), Creatinine (continuous data), BUN (continuous data), hs-CRP (continuous data), cigarette smoking (yes or no), and alcohol drinking (yes or no).

BMI, body mass index; FBG, fasting blood glucose; FINS, fasting insulin; HOMA-IR, homeostasis model assessment of insulin resistance; LDL cholesterol, low-density lipoprotein cholesterol; HDL cholesterol, high-density lipoprotein cholesterol; BUN, blood urea nitrogen; hs-CRP, high-sensitivity C-reactive protein. The bold values indicate the data with a significant P value (P<0.05).

## Discussions

Our results showed that the lumbar BMD was negatively associated with HU in obesity. However, such findings only existed in men, rather than women. On the contrary, no significant relationship between hip BMD and HU was obtained.

Generally speaking, the relationship between BMD and HU is conflicting. Several studies found a positive association between BMD and HU in postmenopausal and older women (16, 26–31). Furthermore, a positive association between lumbar spine BMD and serum uric acid (SUA) was confirmed in postmenopausal women, rather than men (32). On the contrary, no significant relationship between lumbar spine BMD and SUA was obtained in US participants over 30 years (33). Therefore, the relationship between BMD and HU may vary depending on the population. In consideration of the special characteristics of obesity, it is necessary to be concerned about such issues in the context of obesity. It was reported that obesity was associated with a lower level of BMD (21) and a higher prevalence of HU (2), which might partly contribute to the inverse relationship between BMD and HU in obesity (especially the BMI in this population is extremely high: 35.2 ± 5.2). Interestingly, such findings were not found in women, which was in contrast to the previous evidence regarding postmenopausal and older women. It should be noted that the women in our study are relatively young (27.7 ± 9.2 years old). It is reported that estrogen is associated with a higher level of BMD and a lower level of serum uric acid. In this condition, a higher level of estrogen in our women population may contribute to a potential negative relationship between BMD and HU. The aforementioned positive relationship between BMD and HU regarding postmenopausal and older women may be neutralized in the context of young women. Therefore, a final combined null relationship was obtained in our results. Moreover, the BMI in men is significantly higher than that in women (36.9 *vs.* 33.7). Thus, the potential obesity-derived negative relationship between BMD and HU may be much stronger in men, which may partly contribute to the sex difference results in our study. However, our results should be carefully interpreted due to the limited sample size and potential residual confounding with unmeasured variables. Furthermore, the results regarding the lumbar and hip were totally different. Moreover, the L1 lumbar BMD seems to contribute mostly. Therefore, the location for analysis may be involved in the relationship between BMD and HU. Moreover, the lumbar BMD seems to be more sensitive to reflect the issue of obesity. Nevertheless, our results may be restricted by the nature of the cross-sectional design and limited sample size, and more large prospective studies are still needed.

The underlying mechanism behind the inverse relationship between BMD and HU can be listed as follows. The hydrophobic lipid layer of the cell membrane may interfere with the antioxidant properties of uric acid (it mainly acts in human plasma) (34, 35). The intracellular free oxygen radicals are generated during uric degradation, which further enhances intracellular superoxide generation by interacting with NADPH oxidase. It inhibits osteoblast bone formation and stimulates osteoclast bone resorption (36–38). In addition, uric acid might exert adverse effects on bone health by affecting 25-OH-D (1,25D) and parathyroid hormone (PTH) levels. An inverse correlation between 1,25D concentrations and uric acid has been revealed in the HU model and chronic kidney disorder subjects (39–41). However, this association could be reversed by allopurinol, a drug that lowers uric acid levels (41). Similarly, serum uric acid was positively correlated with PTH levels (39, 42). Furthermore, the proinflammatory cytokines causing acute gout attack, including tumor necrosis factor-α, interleukin-1, interleukin-6, and interleukin-8, promote osteoclast differentiation and increases bone resorption (36). However, the issue of obesity has not been considered in current experimental evidence, which should be addressed by further study.

Our study has several advantages. To begin with, this is the first study on the association between BMD and HU in obesity so far. In addition, our study has specified that the relationship between BMD and HU may be varied depending on the bone location. Finally, our results may appeal to the public to pay more attention to bone health in the obese population: an increased level of BMD may be beneficial to the clinical management of obesity. However, the limitations of our study should also be acknowledged. First, the cross-sectional design precludes causal relationships, and further prospective studies are still needed to clarify the concerns. Second, the questionnaire surveys are used to collect some clinical data (smoking and alcohol drinking status, etc.). The potential recall bias cannot be excluded. Third, the sample size of the obese subjects is relatively small, which may inevitably influence the reliability of our results. Fourth, the included subjects are relatively young. Therefore, our results may not reflect the issue at all ages. Finally, some residual or unmeasured confounder remains possible.

In conclusion, our results showed that the lumbar BMD was negatively associated with HU in obesity. However, such findings only existed in men, rather than women. In addition, no significant relationship between hip BMD and HU was obtained. Further large well-designed prospective studies are still needed.

## Data availability statement

The raw data supporting the conclusions of this article will be made available by the authors, without undue reservation.

## Ethics statement

The studies involving human participants were reviewed and approved by The Medical Ethics Committee of Xiangya Hospital, Central South University (202103043). The patients/participants provided their written informed consent to participate in this study.

## Author contributions

MW and YiZ decided and conceptualized this article and revised the draft. YiZ and MT wrote the manuscript. MT and JW collected and analyzed the data. YiZ and JW prepared the figures and tables. MW and JW were the guarantors of the overall content. All authors contributed to the article and approved the submitted version. 
